# Supplementary material for: Potential endocrine disrupting properties of toys for babies and infants
Source: PLoS One. 2020 Apr 3;15(4):e0231171. doi: 10.1371/journal.pone.0231171 (PMC7122770; doi:10.1371/journal.pone.0231171)
Supplement: S1 Table — (DOC) [file pone.0231171.s001.doc]

**S1 Table: Data from the analysis of detected pure substances in ER-CALUX® (data for Figure 2)**

| **Concentration in bioassay medium [mol/l]** | **% E2 max** | | | | | | | | | |
| --- | --- | --- | --- | --- | --- | --- | --- | --- | --- | --- |
|  | **benzophenone** | | **diethylphthalate** | | **bisphenol A** | | **tributyl citrate** | | **17β-estradiol** | |
|  | **mean** | **std dev** | **mean** | **std dev** | **mean** | **std dev** | **mean** | **std dev** | **mean** | **std dev** |
| 1E-13 |  |  |  |  |  |  |  |  | 4% | 1% |
| 3E-13 |  |  |  |  |  |  |  |  | 5% | 1% |
| 6E-13 |  |  |  |  |  |  |  |  | 6% | 1% |
| 1E-12 |  |  |  |  |  |  |  |  | 7% | 3% |
| 2E-12 |  |  |  |  |  |  |  |  | 13% | 1% |
| 3E-12 |  |  |  |  |  |  |  |  | 19% | 3% |
| 1E-11 |  |  |  |  |  |  |  |  | 45% | 10% |
| 3E-11 |  |  |  |  |  |  |  |  | 85% | 7% |
| 1E-10 |  |  |  |  |  |  |  |  | 98% | 9% |
| 5E-10 |  |  |  |  |  |  |  |  | 100% | 8% |
| 5,00E-09 |  |  |  |  | 5% | 1% |  |  |  |  |
| 5,00E-08 |  |  |  |  | 11% | 1% |  |  |  |  |
| 5,00E-07 |  |  |  |  | 116% | 16% |  |  |  |  |
| 2,50E-06 | 7% | 2% | 9% | 1% | 151% | 12% | 4% | 0% |  |  |
| 5,00E-06 | 14% | 3% | 10% | 1% | 154% | 24% | 6% | 1% |  |  |
| 5,00E-05 | 57% | 12% | 23% | 2% |  |  | 34% | 4% |  |  |
| 2,50E-04 | 101% | 11% | 47% | 4% |  |  | 8% | 4% |  |  |
| 5,00E-04 | 122% | 7% | 69% | 12% |  |  | 3% | 0% |  |  |

% E2 max………... relative luciferase activity compared to the highest signal of the reference standard 17β-estradiol (E2)

Mean……………… arithmetic mean of an analysis in triplicates

Std dev…………… standard deviation of an analysis in triplicates
